# Correction: A New Bacillus anthracis Found in Wild Chimpanzees and a Gorilla from West and Central Africa

**DOI:** 10.1371/journal.ppat.0020064

**Published:** 2006-06-30

**Authors:** Fabian H Leendertz, Saniye Yumlu, Georg Pauli, Christophe Boesch, Emmanuel Couacy-Hymann, Linda Vigilant, Sandra Junglen, Svenja Schenk, Heinz Ellerbrok

DOI: 10.1371/journal.ppat.0020008

In *PLoS Pathogens,* vol 2, issue 1:

The data upon which Figures 1 and 2 are based had not been deposited in a publicly available repository at the time of publication. Readers can now access the data at Genbank (http://www.ncbi.nlm.nih.gov/entrez/query.fcgi?db=Nucleotide). The accession numbers are DQ497165–DQ497182.

